# *Aspergillus nidulans*: A Potential Resource of the Production of the Native and Heterologous Enzymes for Industrial Applications

**DOI:** 10.1155/2020/8894215

**Published:** 2020-08-01

**Authors:** Amit Kumar

**Affiliations:** Department of Biotechnology, College of Natural and Computational Sciences, Debre Markos University, Debre Markos, Ethiopia

## Abstract

*Aspergillus nidulans* is a filamentous fungus that is a potential resource for industrial enzymes. It is a versatile fungal cell factory that can synthesize various industrial enzymes such as cellulases, *β*-glucosidases, hemicellulases, laccases, lipases, proteases, *β*-galactosidases, tannases, keratinase, cutinases, and aryl alcohol oxidase. *A. nidulans* has shown the potential to utilize low-cost substrates such as wheat bran, rice straw, sugarcane bagasse, rice bran, coir pith, black gram residue, and chicken feathers to produce enzymes cost-effectively. *A. nidulans* has also been known as a model organism for the production of heterologous enzymes. Several studies reported genetically engineered strains of *A. nidulans* for the production of different enzymes. Native as well as heterologous enzymes of *A. nidulans* have been employed for various industrial processes.

## 1. Introduction

Enzymes have various kinds of industrial applications including pulp and paper, laundry, food, animal feed, brewery and wine, textile, and bioenergy industry [1–3]. Commercial production of enzymes had a market of $4.9 billion in 2015 and an expected annual growth rate of 4.7% for 2016–2021 [4, 5]. Cost-effective and efficient industrial manufacturing is achieved with the utilization of enzymes when compared to chemical catalysts. Enzymes are highly specific and environmentally friendly and can work under mild conditions [2, 6]. Enzymes are produced by several microorganisms such as bacteria, actinomycetes, and fungi, but fungi are of great interest because they secrete extracellular enzymes in higher quantity [7]. Filamentous fungi, including Aspergilli, are widely used for industrial production of enzymes [4]. *A. nidulans* is a versatile fungal resource that has shown the potential for the production of different enzymes including cellulase [8], *β*-glucosidase [9], xylanase [10], laccase [11], lipase [12], protease [13], *β*-galactosidase [14], tannase [15], keratinase [16], cutinase [17], and aryl alcohol oxidase [4]. This review is focused on the potential of wild strains of *A. nidulans* for native enzyme production and the capability of expression of heterologous enzymes.

Fungi are used in industrial protein production due to their excellent ability to secrete a large quantity of proteins [18]. Heterologous protein expression in filamentous fungi has been extensively studied due to their natural ability to produce a large quantity of hydrolytic enzymes for industrial processes at large scale. In a general protein expression, a selected gene that encodes the target enzyme is expressed in a fungal cell factory such as *Aspergillus niger*, *Aspergillus oryzae*, *Trichoderma reesei*, and *Aspergillus nidulans* [2, 19]. Filamentous fungi belonging to the genus *Aspergillus* are commonly associated with biomass degradation and produce a wide range of hydrolases. *Aspergillus* spp. are considered suitable host organisms for industrial enzyme production due to their high secretion capacity, GRAS (generally regarded as safe) status, rapid growth on inexpensive media, and a large range of native enzymes produced [20]. *A. nidulans* has been established as a model organism for the secretion of a large quantity of heterologous enzymes. It is a well-recognized filamentous fungus that is widely used for bioengineering research. Its physiology, morphology, and optimal growth conditions are well characterized [21–23]. In the last few years, several investigations have been performed on *A. nidulans* as a host organism for the production of different heterologous enzymes such as lipase [22], xylanase [23], xyloglucanase (GH12) [24], endoxylanase (GH11) [25], arabinofuranosidase (GH54) [25], and aryl alcohol oxidase [4].

**Figure 1:**
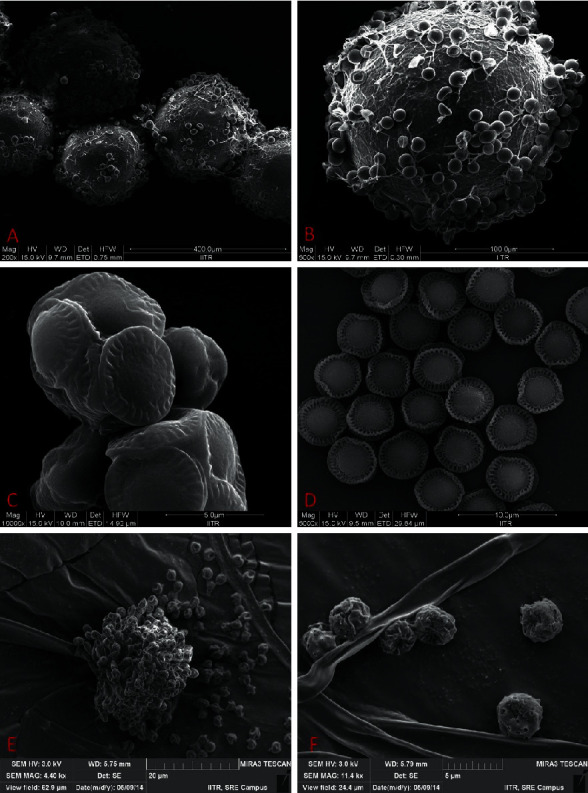
Sexual and asexual structures of *A. nidulans* AKB-25: (a) Cleistothecia, (b) close-up of peridium of cleistothecium with hulle cells, (c) developing ascus, (d) ascospores, (e) conidiophores, and (f) conidia [26].

The life cycle of *A. nidulans* progresses through both sexual and asexual reproduction stages. These are controlled by diverse environmental factors such as light, oxygen, and nutrient availability. Generally, sexual reproduction starts after asexual conidiation and encouraged by oxygen limitation and lack of light. *A. nidulans* forms a closed fruiting body known as cleistothecium (Figures 1(a) and 1(b)) [27]. Hulle cells are characteristic structures normally produced by some species of the genus *Aspergillus.* These cells are found in the association of cleistothecia of all ascogenous species of the *Aspergillus nidulans* group (Figure 1(b)) [28].

Classification is as follows:

  Kingdom: Fungi
  Phylum: Ascomycota
  Class: Eurotiomycetes
  Order: Eurotiales
  Family: Trichocomaceae
  Genus: *Aspergillus*
  Species: *nidulans* [12]

## 2. Enzyme Production by *A. nidulans*

Enzyme production through fungi has been carried out under solid-state fermentation (SSF) and submerged fermentation (SmF). SmF has been given more attention due to easy control and measurement of fermentation parameters, reduced fermentation time, and basic methods to harvest and purify enzyme products [2, 29]. In recent years, intensive research has been performed on SSF and it has earned credibility due to its low water consumption, fewer contamination chances, low energy requirement, and high product yield [9, 26]. Although *A. nidulans* have shown potential for enzyme production under both SSF and SmF, most studies report enzyme production by *A. nidulans* under SmF (Table 1). The fermentation parameters such as fermentation medium, duration, pH, and temperature affect enzyme production under SSF and SmF. The moisture content is a critical parameter under SSF while shaking or static conditions are crucial during SmF [39] (Table 2). The availability of a suitable nitrogen source and the supplementation of surfactants are also essential factors that determine enzyme synthesis. The development of a suitable medium for microbial growth can potentially reduce medium cost and improves enzyme production.

### 2.1. Utilization of Low-Cost Substrate for Enzyme Production

Enzyme production is dependent on the nature of carbon sources, favorable degradability, bare chemical composition, physical associations, accessibility of substrate, and presence of some nutrients [9, 35]. The higher production cost of an enzyme makes the final product expensive. Therefore, economic alternates for enzyme production are in the focus of researchers. During the fermentative production of enzymes, the substrate cost has been considered up to 30% of total production cost. The use of agricultural residues or other wastes as a substrate represents a viable option for the cost-effective production of enzymes [1, 44]. Several agricultural residues or wastes have shown potential for the induction of various kinds of enzymes by *A. nidulans*.

#### 2.1.1. Wheat Bran

Wheat bran has been proved a suitable substrate for enzyme production due to several reasons. Wheat bran is a rich source of fiber, minerals, vitamins, and phenolic compounds [45]. It is a suitable source of nitrogen compared to other agroresidues. Wheat bran has been reported having a minimum *C*/*N* ratio that is favorable for enzyme production [26]. Wheat bran remains loose during moist conditions and, therefore, provides a large surface area for fungal growth and enzyme production [26, 46]. The worldwide availability of wheat bran also makes it a potential substrate for enzyme production. Worldwide wheat production is estimated at 690 million tonnes annually. The weight ratio of bran to milled wheat is about 25 : 75. Hence, wheat bran generation is estimated at 150 million tonnes all over the world. So, the properties and availability of wheat bran represent it as a potential substrate for enzyme production [43, 45]. Taneja et al. [10] studied the production of alkaline and thermostable xylanase by *A. nidulans* KK-99 and used wheat bran as a substrate for xylanase production (40 IU/ml) under SmF [10].

Wheat bran was employed as a low-cost substrate by *Emericella nidulans* NK-62 for xylanase production. Czapek's mineral solution supplemented with 2% wheat bran resulted in 362 IU/ml of xylanase under SmF after seven days of incubation [31]. During cocultivation of *A. nidulans* AKB-25 and *Penicillium* sp. AKB-24, 50% wheat bran and 50% black gram residue were used as a substrate for cellulase and xylanase production under SSF conditions. 4.04, 203.70, 3.70, 35.28, and 3674.94 IU/gds of FPase, endoglucanase, exoglucanase, *β*-glucosidase, and xylanase, respectively, were produced by cocultivation of *A. nidulans* AKB-25 and *Penicillium* sp. AKB-24 [26].

#### 2.1.2. Sugarcane Bagasse

Bagasse is the main residue that remains after sap extraction from sugarcane in the sugar industry. The processing of each tonne of sugarcane produces 740 kg of juice and 260 kg of moist bagasse. Sugarcane bagasse is an abundant resource with easy accessibility, cost-effectivity, and compressibility [35, 47]. Therefore, bagasse can be a renewable resource for enzyme production by *A. nidulans*. Jabasingh and Nachiyar [35] utilized sodium hydroxide pretreated sugarcane bagasse for cellulase production by *A. nidulans* MTCC344 using response surface methodology under SSF conditions. The maximum endoglucanase activity (28.84 IU/gds) was observed at the moisture content of 60% and a temperature of 40°C [35].

#### 2.1.3. Rice or Paddy Straw

Rice straw is exploited as a substrate for the cellulolytic and hemicellulolytic enzymes. Globally, about 744.995 million metric tonnes of rice were produced during the year 2013-2014. Rice crop produces rice straw with a ratio of 1 : 1.5 for rice grain to rice straw. So, the huge generation of rice straw can be a cost-effective substrate for enzyme production [48, 49]. Bagga and Sandhu [33] utilized different carbon sources for cellulase production by *A. nidulans* and observed that delignified bagasse, paddy straw, and wheat straw resulted in higher production of cellulase compared to carboxymethyl cellulose, xylan, pectin, lactose, and glycerol. Among agroresidues, tested paddy straw produced maximum cellulase [33]. da Silva Mennezes et al. [23] carried out SSF of rice husk by *Aspergillus nidulans* XynC A773 and produced 187.9 U/g of xylanase. The xylanase production was influenced by fermentation parameters such as basal medium, moisture content, and interactions between granulometry and inoculum size [23].

#### 2.1.4. Black Gram Residue

Black gram is a pulse crop that is grown in Asian countries over an area of 3.10 million hectares, and the annual production of black gram is 1.40 million tonnes. The harvesting of crops generates a huge amount of black gram residue that might be a resource for the production of microbial enzymes [9, 50]. Kumar et al. [9] performed SSF for cellulase and xylanase production by *A. nidulans* AKB-25 using black gram residue as a substrate. Untreated black gram residue resulted in the maximum endoglucanase (152.14 IU/gds), FPase (3.42 FPU/gds), and xylanase (2491.41 IU/gds) activities with the moisture content of 80% and 4 days of the fermentation period.

#### 2.1.5. Coir Pith

Coconut pith or coir pith is a natural renewable resource that is a by-product of the fiber extraction process. The extraction of 1 kg of coir fiber generates 2 kg of coir pith and it contains approximately 25–29% of cellulose, 31% lignin, and 15% hemicelluloses. Coir pith has no economic value; it is dumped outside the coir industry in a huge amount. It has high ash content and poor combustion properties and emits high smoke when burnt. The high lignin content makes it unsuitable as the raw organic manure for crops [30, 51–53]. Therefore, the utilization of coir pith for enzyme can solve the problems associated with coir pith disposal. *A. nidulans* were isolated from degraded coir retting yarns of backwaters and used for cellulase production using pretreated coir pith as a substrate under SSF conditions. The maximum endoglucanase activity of 28.64 U/gds was observed at the moisture level of 70% and temperature 40°C with pH 5.0 [30].

#### 2.1.6. Rice Bran

Rice bran is the by-product of the rice milling process with worldwide production of 50–60 million tonnes each year which is used as animal feed and pollutant adsorbent and for the production of rice bran oil. The amount of rice bran generated is for the excess of its local applications, thereby frequently leading to disposal problems [54–56]. It contains a substantial amount of proteins and oil; therefore, it is a potential substrate for lipase and protease production. Niaz et al. [12] tested different substrates such as wheat bran, rice bran, brassica bran, almond meal, and mustard meal for extracellular lipase production under SmF. Among all substrates, rice bran was found to induce maximum lipase (33.3 U/ml/min) by *A. nidulans* (Mbl-S-6).

#### 2.1.7. Chicken Feather

Poultry houses generate a huge amount of feather waste that is an abundant keratinous material with 91% of *β*-keratin. It has been reported that daily accumulation of feather waste reached 5 million tonnes around the world. Generally, it is disposed of in dumps, land-filled, or burnt in incinerators that produce huge environmental pollution [57, 58]. Therefore, the utilization of feather waste as a substrate can minimize enzyme production cost and environmental pollution. Saber et al. [16] used the powder of chicken feather waste as the sole source of carbon and nitrogen for protease production by *A. nidulans* K7 (55.8 U/ml) under SmF conditions.

### 2.2. Effect of Nitrogen Sources on Enzyme Production

Fungal growth and synthesis of extracellular enzymes are influences by the availability of precursors of protein synthesis. The suitable combination nitrogen source, carbon source, and fungal strain are necessary for optimum maximum production of an enzyme [26, 39]. Filamentous fungi use various compounds as nitrogen sources. However, the ability of fungi to use different nitrogen sources is selective and preferable which is controlled by complex regulation systems at the transcriptional level [2, 59]. Various organic and inorganic nitrogen sources have tested for the production of different enzymes by *A. nidulans.* Liu et al. [2] developed a cost-effective medium for the production of aryl alcohol oxidase (AAO) using *A. nidulans* under SmF. Several nitrogen sources were tested for AAO production and inorganic nitrogen sources; namely, sodium nitrate and potassium nitrate were found better sources compared to peptone, yeast extract, urea, ammonium sulfate, ammonium nitrate, and ammonium chloride. Corn steep liquor (CSL) was observed most effectively among all tested nitrogen sources [2]. Extracellular lipase production by *A. nidulans* (Mbl-S-6) was performed using different nitrogen sources such as peptone, urea, ammonium sulfate, ammonium chloride, and ammonium molybdate. Among all tested sources, ammonium sulfate resulted in maximum lipase production [12]. The effect of organic and inorganic nitrogen sources on lipase production by *E. nidulans* NFCCI 3643 was studied by Latha et al. [37], and yeast extract resulted in maximum lipase production among tested organic nitrogen sources. But, ammonium sulfate caused the maximum lipase production among all tested organic and inorganic nitrogen sources. Kamran et al. [14] tested various organic and inorganic nitrogen sources for *β*-galactosidase production and noticed that peptone (0.5%), yeast extract (0.5%), ammonium sulfate (0.2%), and ammonium nitrate (0.2%) improved the production of *β*-galactosidase by *A. nidulans* under SmF conditions [14]. The effect of various nitrogen sources on protease production by *A. nidulans* was studied, and peptone induced maximum protease production (1129.8 U/ml) compared to yeast extract (868.0 U/ml), malt extract (805.0 U/ml), ammonium sulfate (141.4 U/ml), ammonium nitrate (302.4 U/ml), and ammonium chloride (488.6 U/ml) under SmF [13]. Ali and Saad El-Dein [60] studied the effect of three nitrogen sources on the production of cellulase enzyme complex by *A. nidulans* and observed sodium nitrate as the most effective nitrogen source for endoglucanase, avicellase, and *β*-glucosidase production.

### 2.3. Effect of Surfactants Supplementation

The stimulatory effect of surfactants on enzyme production is well established. Surfactants improve the penetration of water molecules into a solid substrate matrix that increases the swelling of the substrate leading to improved available surface area for microbial growth [61, 62]. Surfactants increase the permeability of the cell membrane that affects the secretion of certain proteins. Moreover, surfactants are also found to assist the release of cell-bound enzymes into fermentation media [62–64]. Kumar et al. [9] supplemented SSF production media with surfactants, namely, Tween-20, Tween-40, Tween-60, Tween-80, Triton-X-100, SDS, and EDTA. Among all tested surfactants, Tween-20, Tween-40, Tween-60, Tween-80, and Triton-X-100 showed the stimulatory effect on cellulase and xylanase production by *A. nidulans* AKB-25 while SDS and EDTA showed an inhibitory effect on enzyme production. 0.05% (w/v) of Triton-X-100 resulted in maximum FPase and endoglucanase activity while maximum xylanase production was caused by the supplementation of 0.1% (w/v) Tween-80 [9]. In another study, several surfactants including gum Arabic, SDS, PEG, Tween-80, and Triton-X-100 were tested for lipase production. Gum arabic enhanced lipase production compared to control while other surfactants decreased lipase production by *E. nidulans* NFCCI3643 [37].

## 3. *A. nidulans*, an Ideal Fungus for Expression of Heterologous Enzymes

Filamentous fungi are important resources for large-scale enzyme production. However, the production level is low and this limitation stimulates the research on gene manipulations [65]. The heterologous expression is reported as an alternative to increase the production of enzymes. Improved production of enzymes makes industrial processes cheaper especially when coupled to low-cost inducer substrates [22]. *A. nidulans* is an excellent model fungal system with standardized techniques for genetics and biochemical characterization of any phenotype of interest. It has been extensively studied for genetic manipulation for enhanced synthesis or secretion of different types of macromolecules. *A. nidulans* is amenable to molecular techniques, and its complete genome has been sequenced [38, 66].

A high-expression secretion vector (pEXPYR) directing overproduction of client proteins was engineered. This vector directs proteins towards extracellular medium in *A. nidulans* A773 and *A. awamori* ATCC22342. This upgraded expression/secretion system was used to generate a core set of hyperproducing hemicellulolytic enzymes. The serial integration of multiple client genes enables the production of a cocktail of enzymes within a single expression host [18]. *A. nidulans* A773 was used for the simultaneous production of xylanase and the liberation of xylooligosaccharide using rice husk as a substrate [23]. Endoxylanase and arabinofuranosidase encoding genes from *Penicillium funiculosum* and *Aspergillus niger*, respectively, were transferred into model organism *A. nidulans.* Hypersecretion of endoxylanase (301.2 U/mg) and arabinofuranosidase (115.55 U/mg) by *A. nidulans* was observed using the expression medium having pyridoxine and 2% maltose as inducer at 37°C. Endoxylanase and arabinofuranosidase activities were assayed against rye arabinoxylan and sugar beet arabinan, respectively, as substrate [25]. Abdella et al. [67] modified *A. nidulans* by the integration of an AFUMN-GH10 gene from *Aspergillus fumigatus* var. *niveus* for endo-*β*-1,4-xylanase production. This genetically engineered *A. nidulans* resulted in higher secretion of xylanase in the presence of maltose. A 2-level Plackett-Burman design and 3-level Box-Behnken design were used for the optimization of nutrient medium components, and xylanase activity of 1620 U/ml (12460 U/g of maltose) was attained using an optimum medium. This xylanase activity was 280% higher than the maximum activity with the initial basic medium [67]. A xyloglucanase (AtXEG12) from *Aspergillus terreus* was expressed in *A. nidulans* and purified. The engineered *A. nidulans* having the AtXEG12 gene was grown under SmF and SSF in an air-lift bioreactor and a column type bioreactor, respectively. During SmF, the highest enzyme activity (0.45 IU/ml) was observed after 24 h while a maximum enzyme activity of 3.15 IU/ml was found during SSF conditions [24].

Acidic, thermostable endoglucanase from *A. nidulans* was expressed in *Pichia pastoris*, and purification and biochemical characterization of the recombinant enzyme were carried out. The maximum endoglucanase activity was observed at temperature 50°C and pH 4.0. The recombinant endoglucanase retained 100% activity when incubated at 45 and 55°C for 72 h. Recombinant endoglucanase was assayed for the capacity of hydrolyzing natural substrates, namely, banana stem, ball-milled steam-exploded sugarcane bagasse, soybean residue, and corn stover [68]. *Beauveria bassiana* lipase gene bbl1 sequence was cloned into the expression vector pExpyr in-frame with *Aspergillus niger* glucoamylase signal peptide resulting in a plasmid named as pExpyr + bbl1. *A. nidulans* A773 was transformed using this vector. A variety of carbon sources including cassava peels, corn syrup, sorghum seed, and wheat bran were used to improve recombinant lipase production. Monosaccharides such as fructose and glucose enhanced lipase production in combination with previously described carbon sources especially sorghum seeds and wheat bran [22]. *A. nidulans* A773 was genetically modified and overexpresses and secretes *Myceliophthora thermophila* aryl alcohol oxidase (AAO) in maltose containing media. This mutant strain of *A. nidulans* is unable to synthesize its pyridoxine. Pyridoxine limitation can be used to control cell growth, diverting substrate to protein synthesis. In agitated fermentation, AAO production was found similar when media were with 1 mg/L pyridoxine and without pyridoxine. However, the treatment lacking pyridoxine had to be supplemented with pyridoxine after 156 h of fermentation to sustain continued enzyme production. The use of extremely diluted pyridoxine levels allowed reduced fungal growth while maintaining steady enzyme production [69].

## 4. Applications of *A. nidulans* Enzymes

*A. nidulans* can synthesize a variety of industrial enzymes including cellulases, xylanases, laccases, lipases, proteases, *β*-galactosidases, tannases, keratinases, cutinases, and aryl alcohol oxidases (Table 1). These enzymes find applications in various industries such as food, brewery, and wine, biofuels, animal feed, textile and laundry, and pulp and paper.

### 4.1. Hydrolysis of Lignocellulosic Biomass

Lignocellulosic biomass stores a huge amount of carbohydrates including cellulose and hemicelluloses. The hydrolysis of these carbohydrates is one of the key steps in the production of biofuels and value-added products from lignocellulosic biomass. The hydrolysis can be performed by acid hydrolysis or enzymatic hydrolysis. Enzymatic hydrolysis has been proved to have several advantages such as high yield of monomer sugars, mild reaction conditions, and negligible by-products formation [70]. The enzymatic hydrolysis of alkaline pretreated pearl millet stover was performed by a multienzyme from *A. nidulans* AKB-25 and obtained a saccharification yield of 64.77% at an enzyme dose of 15 FPU/g of the substrate with the addition of 0.15% of Tween-80 as a surfactant. The crude enzyme efficiency might be due to the fact that 1 FPU of the enzyme contained 39.39 IU of endoglucanase, 11.68 IU of *β*-glucosidase, and 642.47 IU of xylanase [71]. Plant polysaccharide saccharification was improved by the cooperation of *A. nidulans* enzymes. Two endoxylanases and a *β*-xylosidase cooperate in the hydrolysis of beechwood xylan. The two endoxylanases act on different linkages in beechwood xylan, since the mixture of two enzymes provides a higher level of enzymatic hydrolysis. The positive synergy was found with a *β*-xylosidase, with the highest hydrolysis efficiency when all three enzymes were utilized together. Two endoglucanases, a *β*-glucosidase, and an *α*/*β*-glucosidase cooperated during the hydrolysis of *β*-glucan [72]. Cocultivation of four *A. nidulans* recombinant strains was used for the production of a cocktail of enzymes having GH51 arabinofuranosidase, GH11 endo-1,4-xylanase, GH43 endo-1,5-arabinanase, and GH12 xyloglucan specific endo-*β*-1,4-glucanase. This set of recombinant enzymes was used as an alternative to alkaline sugarcane bagasse pretreatment. This enzymatic was more efficient than the alkaline method in maintaining cellulose integrity and exposing this cellulose to the subsequent saccharification process [73].

### 4.2. Biodiesel Production

Cutinases are versatile carboxylic esters that catalyze the breakdown of both small-molecule esters and polyesters. They have great potential in biodiesel production. Sesame oil was used as a substrate for fatty acid methyl esters (biodiesel) production by cutinase from *A. nidulans*. After 72 h of incubation, 63 and 2% yield of fatty acid methyl esters was obtained by chemical and cutinase treatment, respectively. The low yield by cutinase treatment was observed due to the inhibition of enzyme activity by methanol and glycerol that are present in the reaction mixture. This might be improved by reducing the inhibitory effect and optimizing the reaction parameters [74]. Gonçalves et al. [22] studied biodiesel production from Acai (*Euterpe oleracea* Martius), Buriti *(Mauritia flexuosa)*, and coffee grounds oils using recombinant lipase produced by *A. nidulans*. Transesterification reaction of coffee spent, Buriti, and Acai oils by immobilized lipase in Sepabead-C18 in the presence of ethanol was performed [23].

### 4.3. Pulp and Paper Industry

Microbial enzymes present an environment-friendly alternative to conventional chemical processes. Microbial enzymes such as cellulases, xylanases, pectinases, and amylases are applicable for the different processes such as biobleaching, deinking of waste paper, and enzyme-assisted refining. [75]. Alkaline active xylanase produced by *A. nidulans* KK-99 was tested and used for biobleaching of kraft pulp. The xylanase dose of 1.0 IU/g of dry pulp showed optimum bleaching effect at pH 8.0 and a temperature of 55°C for 3 h reaction time [10]. In another recent study, thermo- and alkali-tolerant xylanase from *A. nidulans* was reported for bleach-boosting of bamboo pulp. The maximum biobleaching efficiency was found at an enzyme dose of 20 U/g of dry pulp with an incubation time of 4 h. The release of phenolic and hydrophobic compounds was increased by approximately 8 and 22 times, respectively, at the end of the 6 h reaction period [76]. Kumar et al. [9] studied cellulase and xylanase production by *A. nidulans* AKB-25 and used the enzyme for biodeinking of mixed-office waste (MOW) paper. Pulp freeness, brightness, and deinking efficiency of MOW paper were improved by 34.47, 4.6, and 25.01%, respectively, and reduced dirt count by 74.70% compared to control. Physical strengths such as tensile index, burst index, and double-fold number were also improved during the biodeinking of MOW [9].

### 4.4. Pharmaceutical Products

Enzymatic hydrolysis of xylan rich agricultural residues produces xylooligosaccharides (XOs) that are considered as probiotic. XOs are sugar oligomers that are composed of *β*-1,4-linked xylose units released by xylan degradation. The presence of *β*-1,4-linkage in XOs makes them resistant to hydrolysis compared to *α*-linkage that is digested easily by human digestive enzymes. XOs have the advantage to be fermented by specific bacteria such as *Bifidobacterium* and *Lactobacillus* that act as probiotics [77–79]. SSF of rice husk was carried out by *A. nidulans* XynC A773 to produce xylanase and XOs. *A. nidulans* XynC A773 produced 23.9 mg/g of the substrate under SSF that is more concentrated and can be extracted easily compared to the diluted medium of submerged culture [23]. Gonçalves et al. [25] studied the production of XOs from pretreated sugarcane bagasse by the synergic action of GH11 endoxylanase and GH54 arabinofuranosidase produced by *A. nidulans* [25]. The genetically modified strain of *A. nidulans* A773 produced xylanase and arabinofuranosidase using soybean fiber as a substrate under SSF, and these enzymes were subsequently exploited to produce XOs from the same agricultural residue. The maximum yield of XOs obtained was 28% (mass fraction of xylan), showing final concentration (in mg·g^−1^ arabinoxylan) of 138.36 xylobiose (X2), 96.96 xylotriose (X3), and 53.04 xylotetraose (X4) in 9 h of reaction time [80].

### 4.5. Dye Decolorization and Industrial Wastewater Treatment

Dye wastewater from textile and dyestuff industries is one of the most difficult wastewater types to treat. Dyes are synthetic compounds with aromatic molecular structures that make them more stable and difficult for biodegradation [81]. Laccases and laccase-mediator systems (LMSs) have great potential for decolorization and detoxification of industrial wastewater. Laccases are multicopper oxidases that can degrade a variety of dyes [82]. Laccases can decolorize wastewaters from olive mills and pulp mills by removing colored phenolic compounds [83]. Crude and purified laccases from *A. nidulans* TTF6 were utilized for decolorization of various dyes including congo red, methyl orange, methyl violet, methyl red, coomassie blue, victoria blue, and bromophenol blue. The crude enzyme required a longer treatment time (7 days) compared to purified one (up to 48 h) at 40°C. Out of the two categories of tested dyes, azo ones (e.g., methyl red) were slowly removed compared to triphenyl amines (e.g., Coomassie blue and victoria blue). Although methyl violet belongs to triphenylamine, it took a long time for decolorization in the presence of *A. nidulans* TTF6 laccase. A higher decolorization rate was observed at 40°C compared to 20°C, obeying general enzyme catalysis characteristics [38]. *Emericella nidulans* var. *nidulans* (anamorph: *Aspergillus nidulans*) was isolated from the sediments of pulp and paper mill that was used for decolorization and detoxification of pulp and paper mill effluent. The treatment process was optimized by the Taguchi approach. The maximum reduction in color (66.66%) and lignin (37%) was observed at temperature (30–35°C), agitation (125 rpm), pH (5.0), and treatment duration of 24 h. The nutrients including dextrose and tryptone were provided at a concentration of 0.25 and 0.1%, respectively [84].

### 4.6. Food Industry

Some strains of *A. nidulans* have been found to produce *β*-galactosidase that is widely used in the different biotechnological processes of the food industry. It converts lactose sugar into *β*-glucose and *β*-galactose that are employed to manufacture sugar syrups. The sensorial properties of food can be improved through the biocatalysis of lactose. This process also enhances the creaminess of ice cream and the texture of various baked products. Furthermore, the enzyme action releases the monosaccharide that improves the sweetness of the product [14, 85, 86].

## Figures and Tables

**Table 1:** Production of industrial enzymes from *Aspergillus nidulans*.

*A. nidulans* strain	Substrate	Type of fermentation	Fermentation conditions				Enzyme (yield)	Reference
			Initial pH	*T* (°C)	*I* (days)	*M* (%)		
*A. nidulans* AKB-25	Black gram residue	SSF	8.0	30	4	80	Endoglucanase (152.14 IU/gds), FPase (3.42 FPU/gds), Xylanase (2441.03 IU/gds) *β*-Glucosidase (35.11 IU/gds)	[9]

*A. nidulans*	Coir pith	SSF	5.0	40	11	70	Endoglucanase (28.64 U/gds) FPase (10.23 U/gds), Cellobiase (4.31 U/gds)	[30]

*A. nidulans* KK-99	Wheat bran	SmF	10.0	37	6	—	Xylanase (40 IU/ml)	[10]

*Emericella nidulans* NK-62	Wheat bran	SmF	—	45	7	—	Xylanase (362 IU/ml)	[31]

*A. nidulans*	Carboxymethylcellulose	SmF	5.0	30	6		Cellulase (39.56 U/ml)	[32]

*A. nidulans*	Paddy straw	SmF	5.0	37	8	—	Endoglucanase (3.5 IU/ml)	[33]

*A. nidulans* SU04	CMC	SmF	3–7	40	8	—	Cellulase (60.54 U/ml)	[8]

*A. nidulans*	Corn cob	SmF	—	30	6	—	Xylanase (220 IU/ml)	[34]

*A. nidulans* MTCC344	Sugarcane bagasse	SSF	4.25	40		60	Endoglucanase (28.96 IU/gds)	[35]

*A. nidulans* (Mbl-S-6)	Rice bran	SmF	7.0	40	3	—	Lipase (102 IU/ml/min)	[12]

*A. nidulans*	Olive oil	SmF	—	30	2	—	Lipase (1121 mU/ml)	[36]

*Emericella nidulans* NFCCI 3643	Olive oil	SmF	6.0	30	4	—	Lipase (140 IU/ml)	[37]

*A. nidulans* LCJ249	Glucose, casein	SmF	7.0	30	6	—	Protease (2262.4 U/ml)	[13]

*A. nidulans* KF974331	Sucrose	SmF	—	30	8	—	Laccase (367.68 U/ml)	[11]

*A. nidulans* TTF6	Olga medium	SmF	6.0	40	5		Laccase (2.4 IU/mg)	[38]

*A. nidulans* (FT10)	Tannic acid	SmF	5.5	35	3	—	Tannase (96.2 U/ml)	[15]

*A. nidulans* K7	Chicken feather powder	SmF	7.5	35	5	—	Keratinase (55.8 U/ml)	[16]

*A. nidulans*	Xylose	SmF	3.0	35	10	—	*β*-Galactosidase (60 U/mg)	[14]

*A. nidulans PW1*	Olive oil	SmF	6.5	37	1	—	Cutinase (1.44 IU/ml)	[17]

T: incubation temperature; I: incubation time of fermentation; M: moisture content in case of SSF.

**Table 2:** Factors affecting enzyme production by *A. nidulans*.

Factors	Description	Reference
Temperature	(i) Temperature is one of the most important fungal-dependent parameters that affect enzyme production. (ii) *A. nidulans* has been reported to synthesize the maximum amount of enzyme 30–45°C.	[26, 39]

Initial pH	(i) Fungal enzyme production is pH specific, and changes in pH affect the production and stability of enzymes. (ii) pH is easily maintained during SmF but it is difficult to control during SSF. The metabolic activities of microorganisms can change the pH of the medium during SSF. It may increase or decrease depending on the production or assimilation of organic acids, respectively. (iii) *A. nidulans* can produce enzymes in acidic, neutral, and alkaline ranges of pH (3.0–10.0) (Table 1).	[1, 40]

Incubation period	(i) The shorter incubation time for maximum enzyme production minimizes the chances of contamination and reduces the production cost. (ii) Different strains of *A. nidulans* have shown variation in incubation time from 1 to 11 days under SSF and SmF (Table 1).	[41, 42]

Initial moisture content (SSF)	(i) The moisture content of the substrate is another important parameter during SSF. (ii) Different microorganisms have a specific requirement in terms of moisture availability for their optimum growth and maximum enzyme production. (iii) *A. nidulans* is found to produce the maximum enzyme yield at initial moisture of 60–80%.	[43]

## Data Availability

All the required data are included in the article.

## References

[1] Bharti A. K., Kumar A., Kumar A., Dutt D. (2018). Exploitation of *Parthenium hysterophorous* biomass as low-cost substrate for cellulase and xylanase production under solid-state fermentation using *Talaromyces stipitatus* MTCC 12687. *Journal of Radiation Research and Applied Sciences*.

[2] Liu E., Li M., Abdella A., Wilkins M. R. (2020). Development of a cost-effective medium for submerged production of fungal aryl alcohol oxidase using a genetically modified *Aspergillus nidulans* strain. *Bioresource Technology*.

[3] Čakar U., Grozdanić N., Pejin B. (2018). Impact of vinification procedure on fruit wine inhibitory activity against *α*-glucosidase. *Food Bioscience.*.

[4] Pardo-Planas O., Prade R. A., Müller M., Atiyeh H. K., Wilkins M. R. (2017). Prevention of melanin formation during aryl alcohol oxidase production under growth-limited conditions using an *Aspergillus nidulans* cell factory. *Bioresource Technology*.

[5] BCC-Research, Global Markets for Enzyme in Industrial Application, BCC Research, Wellesley, MA, USA, 2020, https://www.bccresearch.com/market-research/biotechnology/global-markets-for-enzymes-in-industrial-applications.html

[6] Choi J.-M., Han S.-S., Kim H.-S. (2015). Industrial applications of enzyme biocatalysis: current status and future aspects. *Biotechnology Advances*.

[7] Kumar A., Gautam A., Dutt D. (2015). Screening of fungal resources for the production of cellulases and xylanases. *British Biotechnology Journal*.

[8] Anuradha Jabasingh S., ValliNachiyar C. (2011). Optimization of cellulase production by *Aspergillus nidulans*: application in the biosoftening of cotton fibers. *World Journal of Microbiology and Biotechnology*.

[9] Kumar A., Dutt D., Gautam A. (2016). Production of crude enzyme from *Aspergillus nidulans* AKB-25 using black gram residue as the substrate and its industrial applications. *Journal of Genetic Engineering and Biotechnology*.

[10] Taneja K., Gupta S., Chander Kuhad R. (2002). Properties and application of a partially purified alkaline xylanase from an alkalophilic fungus *Aspergillus nidulans* KK-99. *Bioresource Technology*.

[11] Vivekanandan K. E., Sivaraj S., Kumaresan S. (2014). Characterization and purification of laccase enzyme from *Aspergillus nidulans* CASVK3 from vellar estuary south east coast of India. *International Journal of Current Microbiology and Applied Sciences*.

[12] Niaz M., Iftikhar T., Qureshi F. F., Niaz M. (2014). Extracellular lipase production by *Aspergillus nidulans* (MBL-S-6) under submerged fermentation. *International Journal of Agriculture and Biology*.

[13] Gnanadoss J. J., Devi S. K. (2019). Optimization of nutritional and culture conditions for improved protease production by *Aspergillus nidulans* and *Aspergillus flavus*. *Journal of Microbiology, Biotechnology and Food Sciences*.

[14] Kamran A., Bibi Z., Aman A., Qader S. A. U. (2017). Hyper production of *β*-galactosidase from newly isolated strain of *Aspergillus nidulans*. *Journal of Food Process Engineering*.

[15] Hidayathulla S., Shahat A. A., Alsaid M. S., Al-Mishari A. A. (2018). Optimization of physicochemical parameters of tannase post-purification and its versatile bioactivity. *FEMS Microbiology Letters*.

[16] Saber W. I. A., El-Metwall M. M., El-Hersh M. S. (2010). Keratinase production and biodegradation of some keratinous wastes by *Alternaria tenuissima* and *Aspergillus nidulans*. *Research Journal of Microbiology*.

[17] Castro-Ochoa D., Peña-Montes C., González-Canto A. (2012). ANCUT2, an extracellular cutinase from *Aspergillus nidulans* induced by olive oil. *Applied Biochemistry and Biotechnology*.

[18] Segato F., Damásio A. R. L., Gonçalves T. A. (2012). High-yield secretion of multiple client proteins in *Aspergillus*. *Enzyme and Microbial Technology*.

[19] Hoffmeister D., Keller N. P. (2007). Natural products of filamentous fungi: enzymes, genes, and their regulation. *Natural Product Reports*.

[20] Rose S. H., Zyl W. H. v. (2008). Exploitation *Aspergillus niger* of for the heterologous production of cellulases and hemicellulases. *The Open Biotechnology Journal*.

[21] Frandsen R. J. N., Khorsand-Jamal P., Kongstad K. T. (2018). Heterologous production of the widely used natural food colorant carminic acid in *Aspergillus nidulans*. *Science Reports*.

[22] Cerioni Spiropulos Gonçalves E., Martínez Pérez M., Vici A. C. (2020). Potential biodiesel production from Brazilian plant oils and spent coffee grounds by *Beauveria bassiana* lipase 1 expressed in *Aspergillus nidulans* A773 using different agroindustry inputs. *Journal of Cleaner Production*.

[23] da Silva Menezes B., Rossi D. M., Squina F., Ayub M. A. Z. (2018). Comparative production of xylanase and the liberation of xylooligosaccharides from lignocellulosic biomass by *Aspergillus brasiliensis* BLf1 and recombinant *Aspergillus nidulans* XynC A773. *International Journal of Food Science & Technology*.

[24] Vitcosque G. L., Ribeiro L. F. C., de Lucas R. C. (2016). The functional properties of a xyloglucanase (GH12) of *Aspergillus terreus* expressed in *Aspergillus nidulans* may increase performance of biomass degradation. *Applied Microbiology and Biotechnology*.

[25] Gonçalves T. A., Damásio A. R. L., Segato F. (2012). Functional characterization and synergic action of fungal xylanase and arabinofuranosidase for production of xylooligosaccharides. *Bioresource Technology*.

[26] Kumar A., Gautam A., Dutt D. (2016). Co-cultivation of *Penicillium* sp. AKB-24 and *Aspergillus nidulans* AKB-25 as a cost-effective method to produce cellulases for the hydrolysis of pearl millet stover. *Fermentation*.

[27] Wang C.-L., Shim W.-B., Shaw B. D. (2010). *Aspergillus nidulans* striatin (StrA) mediates sexual development and localizes to the endoplasmic reticulum. *Fungal Genetics and Biology*.

[28] Carvalho M. D. F., Baracho M. S., Baracho I. R. (2002). An investigation of the nuclei of hülle cells of *Aspergillus nidulans*. *Genetics and Molecular Biology*.

[29] Singhania R. R., Sukumaran R. K., Patel A. K., Larroche C., Pandey A. (2010). Advancement and comparative profiles in the production technologies using solid-state and submerged fermentation for microbial cellulases. *Enzyme and Microbial Technology*.

[30] Jabasingh S. A. (2011). Utilization of pretreated coir pith for the optimized bioproduction of cellulase by *Aspergillus nidulans*. *International Biodeterioration & Biodegradation*.

[31] Kango N., Agrawal S. C., Jain P. C. (2003). Production of xylanase by *Emericella nidulans* NK-62 on low-value lignocellulosic substrates. *World Journal of Microbiology and Biotechnology*.

[32] Abasingh S. A., Nachiyar C. V. (2010). A new combinational statistical approach for cellulase optimization in *Aspergillus nidulans*. *Indian Journal of Science and Technology*.

[33] Bagga P. S., Sandhu D. K. (1987). Cellulase formation by *Aspergillus nidulans*. *Journal of Fermentation Technology*.

[34] Dos Reis S., Costa M. A. F., Peralta R. M. (2003). Xylanase production by a wild strain of *Aspergillus nidulans*. *Acta Scientiarum Biological Sciences*.

[35] Jabasingh S. A., Nachiyar C. V. (2011). Utilization of pretreated bagasse for the sustainable bioproduction of cellulase by *Aspergillus nidulans* MTCC344 using response surface methodology. *Industrial Crops and Products*.

[36] Mayordomo I., Randez-Gil F., Prieto J. A. (2000). Isolation, purification, and characterization of a cold-active lipase from *Aspergillus nidulans*. *Journal of Agricultural and Food Chemistry*.

[37] Naveena Lavanya Latha J., Lanka S., Pydipalli M. (2015). Optimization of process variables for extracellular lipase production from *Emericella nidulans* NFCCI 3643 isolated from palm oil mill effluent (POME) dump sites using OFAT method. *Research Journal of Microbiology*.

[38] Sahay S., Chaurse V., Chauhan D. (2020). Laccase from *Aspergillus nidulans* TTF6 showing Pb activation for smaller substrates and dyes remediation in all climates. *Proceedings of the National Academy of Sciences, India Section B: Biological Sciences*.

[39] Yoon L. W., Ang T. N., Ngoh G. C., Chua A. S. M. (2014). Fungal solid-state fermentation and various methods of enhancement in cellulase production. *Biomass and Bioenergy*.

[40] Raimbault M. (1998). General and microbiological aspects of solid substrate fermentation. *Electronic Journal of Biotechnology*.

[41] Gautam A., Kumar A., Dutt D. (2015). Production of cellulase-free xylanase by *Aspergillus flavus* ARC-12 using pearl millet stover as the substrate under solid-state fermentation. *Journal of Advanced Enzymes Research*.

[42] Senthilkumar S., Ashokkumar B., Chandraraj K., Gunasekaran P. (2005). Optimization of medium composition for alkali-stable xylanase production by *Aspergillus fischeri* Fxn 1 in solid-state fermentation using central composite rotary design. *Bioresource Technology*.

[43] Bharti A. K., Kumar A., Kumar A., Dutt D. (2018). Wheat bran fermentation for the production of cellulase and xylanase by *Aspergillus niger* NFCCI 4113. *Research Journal of Biotechnology*.

[44] Yegin S., Buyukkileci A. O., Sargin S., Goksungur Y. (2017). Exploitation of agricultural wastes and by-products for production of *Aureobasidium pullulans* Y-2311-1 xylanase: screening, bioprocess optimization and scale up. *Waste and Biomass Valorization*.

[45] Stevenson L., Phillips F., O’sullivan K., Walton J. (2012). Wheat bran: its composition and benefits to health, a European perspective. *International Journal of Food Sciences and Nutrition*.

[46] Dhillon G. S., Oberoi H. S., Kaur S., Bansal S., Brar S. K. (2011). Value-addition of agricultural wastes for augmented cellulase and xylanase production through solid-state tray fermentation employing mixed-culture of fungi. *Industrial Crops and Products*.

[47] Jorapur R., Rajvanshi A. K. (1997). Sugarcane leaf-bagasse gasifiers for industrial heating applications. *Biomass and Bioenergy*.

[48] Gautam A., Kumar A., Bharti A. K., Dutt D. (2018). Rice straw fermentation by *Schizophyllum commune* ARC-11 to produce high level of xylanase for its application in pre-bleaching. *Journal of Genetic Engineering and Biotechnology*.

[49] Trivedi A., Verma A. R., Kaur S. (2017). Sustainable bio-energy production models for eradicating open field burning of paddy straw in Punjab, India. *Energy*.

[50] IIPR (2011). *Vision 2030*.

[51] Asha P., Divya J., Bright Singh I. S. (2016). Purification and characterisation of processive-type endoglucanase and *β*-glucosidase from *Aspergillus ochraceus* MTCC 1810 through saccharification of delignified coir pith to glucose. *Bioresource Technology*.

[52] Awasthi A., Dhyani V., Biswas B., Kumar J., Bhaskar T. (2019). Production of phenolic compounds using waste coir pith: estimation of kinetic and thermodynamic parameters. *Bioresource Technology*.

[53] Dhyani V., Bhaskar T. (2018). A comprehensive review on the pyrolysis of lignocellulosic biomass. *Renewable Energy*.

[54] Pourali O., Asghari F. S., Yoshida H. (2009). Sub-critical water treatment of rice bran to produce valuable materials. *Food Chemistry*.

[55] Liu C., Hao Y., Jiang J., Liu W. (2017). Valorization of untreated rice bran towards bioflocculant using a lignocellulose-degrading strain and its use in microalgal biomass harvest. *Biotechnology for Biofuels*.

[56] Montanher S. F., Oliveira E. A., Rollemberg M. C. (2005). Removal of metal ions from aqueous solutions by sorption onto rice bran. *Journal of Hazardous Materials*.

[57] Abdel-Fattah A. M., El-Gamal M. S., Ismail S. A., Emran M. A., Hashem A. M. (2018). Biodegradation of feather waste by keratinase produced from newly isolated *Bacillus licheniformis* ALW1. *Journal of Genetic Engineering and Biotechnology*.

[58] Vasileva-Tonkova E., Gousterova A., Neshev G. (2009). Ecologically safe method for improved feather wastes biodegradation. *International Biodeterioration & Biodegradation*.

[59] Tudzynski B. (2014). Nitrogen regulation of fungal secondary metabolism in fungi. *Frontiers in Microbiology*.

[60] Ali U. F., Saad El-Dein H. S. (2008). Production and partial purification of cellulase complex by *Aspergillus niger* and *A. nidulans* grown on water hyacinth blend. *Journal of Applied Sciences Research*.

[61] Vu V. H., Pham T. A., Kim K. (2011). Improvement of fungal cellulase production by mutation and optimization of solid state fermentation. *Mycobiology*.

[62] Gautam A., Kumar A., Dutt D. (2017). Production and characterization of cellulase-free xylanase by *Aspergillus flavus* ARC-12 and its application in pre-bleaching of ethanol-soda pulp of *Eulaliopsis binata*. *Research Journal of Biotechnology*.

[63] Pardo A. G. (1996). Effect of surfactants on cellulase production by *Nectria catalinensis*. *Current Microbiology*.

[64] Irfan M., Nadeem M., Syed Q. (2014). One-factor-at-a-time (OFAT) optimization of xylanase production from *Trichoderma viride*-IR05 in solid-state fermentation. *Journal of Radiation Research and Applied Sciences*.

[65] Zubieta M. P., Gerhardt J. A., Rubio M. V. (2020). Improvement of homologous GH10 xylanase production by deletion of genes with predicted function in the *Aspergillus nidulans* secretion pathway. *Microbial Biotechnology*.

[66] Osmani S. A., Liu H., Hynes M. J., Oakley B. R., Goldman G., Osmani S. (2008). Advances in gene manipulations using *Aspergillus nidulans*.. *Aspergilli: Genomics, Medical Aspects, Biotechnology, and Research Methods*.

[67] Abdella A., Segato F., Wilkins M. R. (2019). Optimization of nutrient medium components for production of a client endo-*β*-1,4-xylanase from *Aspergillus fumigatus* var. *niveus* using a recombinant *Aspergillus nidulans* strain. *Biocatalysis and Agricultural Biotechnology*.

[68] de Pinho Tavares E. Q., Rubini M. R., Mello-de-Sousa T. M. (2013). An acidic thermostable recombinant *Aspergillus nidulans* endoglucanase is active towards distinct agriculture residues. *Enzyme Research*.

[69] Pardo-Planas O., Prade R. A., Wilkins M. R. (2017). High-yield production of aryl alcohol oxidase under limited growth conditions in small-scale systems using a mutant *Aspergillus nidulans* strain. *Journal of Industrial Microbiology & Biotechnology*.

[70] Kumar A., Aggarwal D., Yadav M., Kumar P., Kumar V., Yadav M., Kumar V., Sehrawat N. (2019). Biotechnological conversion of plant biomass into value-added products. *Industrial Biotechnology*.

[71] Kumar A., Dutt D., Gautam A. (2016). Pretreatment and enzymatic hydrolysis of pearl millet stover by multi-enzymes from *Aspergillus nidulans* AKB-25. *Cellulose Chemistry and Technology*.

[72] Tramontina R., Robl D., Maitan-Alfenas G. P., de Vries R. P. (2016). Cooperation of *Aspergillus nidulans* enzymes increases plant polysaccharide saccharification. *Biotechnology Journal*.

[73] Lima M. S., Damasio A. R. de L., Crnkovic P. M. (2016). Co-cultivation of *Aspergillus nidulans* recombinant strains produces an enzymatic cocktail as alternative to alkaline sugarcane bagasse pretreatment. *Frontiers in Microbiology*.

[74] Bermúdez-García E., Peña-Montes C., Castro-Rodríguez J. A., González-Canto A., Navarro-Ocaña A., Farrés A. (2017). ANCUT2, a thermo-alkaline cutinase from *Aspergillus nidulans* and its potential applications. *Applied Biochemistry and Biotechnology*.

[75] Kumar A., Gautam A., Dutt D., Yadav M., Sehrawat N., Kumar P., Kumar V., Singh G., Aggarwa N. (2017). Applications of microbial technology in the pulp and paper industry.. *Microbiology and Biotechnology for a Sustainable Environment*.

[76] Khambhaty Y., Akshaya R., Rama Suganya C., Sreeram K. J., Raghava Rao J. (2018). A logical and sustainable approach towards bamboo pulp bleaching using xylanase from *Aspergillus nidulans*. *International Journal of Biological Macromolecules*.

[77] Hong C., Corbett D., Venditti R., Jameel H., Park S. (2019). Xylooligosaccharides as prebiotics from biomass autohydrolyzate. *LWT*.

[78] Poletto P., Pereira G. N., Monteiro C. R. M., Pereira M. A. F., Bordignon S. E., de Oliveira D. (2020). Xylooligosaccharides: transforming the lignocellulosic biomasses into valuable 5-carbon sugar prebiotics. *Process Biochemistry*.

[79] Singh R. D., Banerjee J., Arora A. (2015). Prebiotic potential of oligosaccharides: a focus on xylan derived oligosaccharides. *Bioactive Carbohydrates and Dietary Fibre*.

[80] Pereira G. F., de Bastiani D., Gabardo S., Squina F., Ayub M. A. Z. (2018). Solid-state cultivation of recombinant *Aspergillus nidulans* to co-produce xylanase, arabinofuranosidase, and xylooligosaccharides from soybean fibre. *Biocatalysis and Agricultural Biotechnology*.

[81] Fu Y., Viraraghavan T. (2001). Fungal decolorization of dye wastewaters: a review. *Bioresource Technology*.

[82] Wang S.-N., Chen Q.-J., Zhu M.-J. (2018). An extracellular yellow laccase from white rot fungus *Trametes* sp. F1635 and its mediator systems for dye decolorization. *Biochimie*.

[83] Mishra A., Kumar S., Bhatnagar A. (2019). Potential of fungal laccase in decolorization of synthetic dyes. *Microbial Wastewater Treatment*.

[84] Singhal A., Thakur I. S. (2009). Decolourization and detoxification of pulp and paper mill effluent by *Emericella nidulans* var. *nidulans*. *Journal of Hazardous Materials*.

[85] Kamran A., Bibi Z., Aman A., Ul Qader S. A. (2019). Purification and catalytic behavior optimization of lactose degrading *β*-galactosidase from *Aspergillus nidulans*. *Journal of Food Science and Technology*.

[86] Rosenberg Z. M.-M. (2006). Current trends of *β*-galactosidase application in food technology. *Journal of Food and Nutrition Research*.

